# Radiofrequency echographic multi-spectrometry and DXA for the evaluation of bone mineral density in a peritoneal dialysis setting

**DOI:** 10.1007/s40520-022-02286-7

**Published:** 2022-11-03

**Authors:** Angelo Fassio, Stefano Andreola, Davide Gatti, Beatrice Bianco, Matteo Gatti, Giovanni Gambaro, Maurizio Rossini, Ombretta Viapiana, Riccardo Negrelli, Giovanni Adami

**Affiliations:** 1 Rheumatology Unit, University of Verona, Policlinico GB Rossi, Piazzale A. Scuro, 37134 Verona, Italy; 2 Nephrology Unit, University of Verona, Verona, Italy; 3 Radiology Unit, University Hospital of Verona, Verona, Italy

**Keywords:** REMS, DXA, Bone mineral density, CKD, Osteoporosis, Peritoneal dialysis

## Abstract

**Background:**

The aim of this real-life cross-sectional explorative study was to compare radiofrequency echographic multi-spectrometry (REMS) with dual-energy X-rays absorptiometry (DXA) in the BMD assessment of patients receiving peritoneal dialysis (PD). Furthermore, we investigated the relationship between lumbar aortic calcifications (AOCs) and the DXA lumbar measurements.

**Methods:**

Consecutive patients referring to the PD clinic of our hospital were included. Lumbar spine and femur scans were acquired with both techniques (including lumbar laterolateral DXA scans). The risk assessment of two fracture risk algorithms (FRAX^®^ and DeFRA^®^) were compared. Cohen’s *k* coefficients were used to assess the inter-technique agreement in the classification of patients as osteoporotic. Lumbar AOCs were estimated semi-quantitatively on laterolateral DXA scans.

**Results:**

41 patients were enrolled. No significant differences were documented between the BMD T-scores measured through DXA or REMS at the femur. At the lumbar spine, the DXA anteroposterior mean *T*-score (− 0.49 ± 1.98) was significantly higher than both the laterolateral DXA (− 1.66 ± 0.99) and the REMS (− 2.00 ± 1.94) measurements (*p* < 0.01 vs both). No significant differences were found between the DXA and REMS fracture risk estimates with both algorithms. The inter-technique Cohen’s *k* coefficient (for the worst *T*-score, any site) was 0.421, *p* < 0.001. The discrepancy between the DXA laterolateral and anteroposterior lumbar *T*-score was positively associated with the AOCs extent and severity (*r* = 0.402, *p* < 0.01).

**Conclusions:**

Our data showed a promising agreement, in a real-life PD setting, between DXA and REMS BMD assessment and in the consequent fracture risk estimation and confirm the AOCs interference on the diagnostic accuracy of lumbar DXA.

**Supplementary Information:**

The online version contains supplementary material available at 10.1007/s40520-022-02286-7.

## Introduction

Chronic kidney disease (CKD) is associated with a wide range of bone mineral and endocrine disturbances known as mineral and bone disease (CKD-MBD), a condition characterised by an increased risk of fragility fractures [1]. The fractures’ impact on morbidity and mortality is especially burdensome among patients on renal replacement therapy (RRT) [2, 3] as well as cardiovascular (CVD) complications [4, 5]. Extensive vascular calcifications, a complication often seen in CKD patients, have been related to low bone mineral density (BMD) in this population [6, 7]. Furthermore, also the specific RRT modality seems to play a role in this troublesome liaison. In terms of fracture risk, peritoneal dialysis (PD) has shown less detrimental effects than haemodialysis (HD) on early BMD changes [8]. On the other hand, the influence of the RRT modality on the development of vascular calcifications is unclear, with recent data suggesting that vascular calcifications might develop more in PD than HD [9].

Dual energy X-rays absorptiometry (DXA) is currently considered the gold-standard for the measurement of BMD in the clinical practice, and the Kidney Disease Improving Global Outcomes (KDIGO) 2017 recommendations suggest BMD testing to assess for fracture risk in CKD patients [1]. However, the DXA technique is affected by significant limitations, such as cumbersome machinery, use of ionising radiations with the necessity of shielded environments, and analytic limitations due to ectopic calcifications [10, 11] or pathologic bone formation [12].

The bone densitometry by radiofrequency echographic multi-spectrometry (REMS) is a novel ultrasound-based technique that can reliably assess BMD at the lumbar spine, femoral neck and total hip. REMS has been already validated in post-menopausal osteoporosis [13] and it has been endorsed as a possible alternative to DXA [14]. However, data on populations affected by CKD-MBD are lacking.

The aim of this real-life cross-sectional explorative study was to compare radiofrequency echographic multi-spectrometry (REMS) with DXA in the BMD assessment of patients receiving peritoneal dialysis (PD). Furthermore, we also explored the fracture risk estimates of two different algorithms when calculated with a DXA and REMS-based T-scores. Finally, we investigated the relationship between the extent and severity of lumbar aortic calcifications (AOCs) and their contribution in the overestimation of the DXA-derived lumbar spine BMD.

## Materials and methods

For this study, we enrolled all the patients referring to the peritoneal dialysis clinic of the Nephrology Unit of our hospital between June and September 2021 who accepted to participate. Given the exploratory nature of the study, no sample size estimation was determined.

The study was conducted within the protocol 1483CESC approved by our local Ethics Committee, in accordance with the 1964 Helsinki declaration and its later amendments or comparable ethical standards. Written informed consent was obtained from all participants included.

### Clinical and laboratory variables

Data on the history of CKD, PD, bone-related medications, and fragility fractures were obtained by interviewing all patients during medical examinations and from the electronic medical records. The start date of CKD diagnosis was established after the observation of kidney damage or glomerular filtration rate < 60 mL/min/1.73 m^2^ for three months or more, irrespective of the cause. Venous blood samples were drawn in the morning after an overnight fast. Measurements of serum calcium, phosphorus, and bone-specific alkaline phosphatase (BAP) were obtained using standard laboratory procedures at the central laboratory.

### Serum bone biomarkers

Venous blood samples were drawn in the morning after an overnight fast. Serum samples were collected from all patients at the time of study recruitment, centrifuged, separated, and stored at −80 °C until measurement. An expert laboratory technician, who was blinded to patients’ clinical details, measured 25-hydroxyvitamin D3 [25(OH)D] and intact parathyroid hormone (PTH). Specifically, 25(OH)D and PTH were measured using the IDS-ISYS Multi Discipline Automated Analyzer (Immunodiagnostic System, Boldon, UK) employing immuno-chemiluminescent technology on the fully automated microplate analyser Personal LAB (Adaltis, Rome, Italy). The intra-assay coefficients of variation (CV), in our laboratory, were 3% for PTH (inter-assay CV 6%), and 6% for 25(OH)D (inter-assay CV 9%).

### DXA

A DXA scan was performed in all patients using the GE Lunar iDXA 194 system (GE Healthcare Lunar, Madison, WI, USA) by a single expert operator (R.N.), who was blinded to patients’ clinical details. The employed DXA scanner underwent daily quality control and regular maintenance for the whole study period.

We obtained BMD measurements expressed as T-scores and Z-scores at both the anteroposterior (AP) lumbar spine (L1–L4) and femur (neck and total hip). Trabecular bone score (TBS) was obtained as well (GE TBS INsight 3.0.3.0). Latero-lateral scans (LL) for BMD measurement were performed at the lumbar spine (L2–L3), with the obtainment of T-scores and Z-scores. A T-score ≤ −2.5 was considered for the diagnosis of densitometric osteoporosis, while a Z-score < −2 was considered for the diagnosis of BMD below the expected range for gender and age.

Vertebral fracture analysis (VFA) was performed in all patients in order to detect the presence of vertebral fractures.

To score the AOCs extent, we used the score described by Kauppila et al. [15] and applied it at the LL lumbar spine scans acquired with DXA. As described in the original paper, lesions were graded as follows: 0, no aortic calcific deposits; 1, small scattered calcific deposits filling less than 1/3 of the longitudinal wall of the aorta; 2, one third or more, but less than two-thirds of the longitudinal wall of the aorta calcified; 3, two thirds or more of the longitudinal wall of the aorta calcified.

The semiquantitative score is applied to both the anterior and posterior walls for each of the four vertebrae (L1–L4), thus giving a final score from 0 to 24. Supplementary Fig. 1, panel A, summarizes the score, while in panel B we report an example from one of the subjects included into the study.

To obtain an estimate of the contribution of the AOCs when measuring BMD with the DXA lumbar spine AP scan, we calculated the difference between the AP T-score and the LL T-score at the lumbar spine.

### REMS

A REMS scan performed by a trained expert operator (M.G.), who was blinded to patients’ clinical details, using EchoStation (Echolight Spa, Lecce, Italy) was obtained for all the patients at the lumbar spine, femoral neck, and total hip, and BMD and T-score measurements were obtained at each site. Given the real-life setting of this study, all reports were included: no patient was excluded from the analysis.

### Fracture risk algorithms

Two different fracture risk assessment tools, the Fracture Risk Assessment Tool (FRAX^®^) [16], and the FRAX-Derived Fracture Risk Assessment (DeFRA^®^), an algorithm derived from FRAX^®^ and based on data on fracture risk in the Italian population [17], was calculated for each patient, with the BMD data obtained from the DXA and REMS.

For the calculation of the FRAX^®^ values, for subjects younger than 40 years old, the age of 40 was selected. In addition, for all patients, the variable n. 10, namely secondary osteoporosis (“disorder strongly associated with osteoporosis”), was selected. The femoral neck BMD for DXA and REMS were considered, and, only for DXA, both the TBS-adjusted and unadjusted values were obtained. For the calculation of the DeFRA^®^) values, for subjects younger than 50 years old, the age of 50 was selected. The worst T-score at either the AP lumbar spine, LL lumbar spine (only for DXA), femoral neck or total hip for both DXA and REMS was entered.

### Statistical analysis

Given the exploratory nature of the study, a sample size of at least 40 subjects was established, primarily based on clinical judgment and practical considerations and not on formal statistical reasoning.

Normality for all variables was tested by Shapiro–Wilk test.

To assess for the inter-technique (DXA vs REMS) agreement for the diagnosis of densitometric osteoporosis at each site and worst site we calculated Cohen’s *k* coefficient. We considered values between 0.21 and 0.40 as fair, 0.41–0.60 as moderate, and 0.61–0.80 as substantial agreement [18].

Intraclass correlation coefficients (ICC) for single and average measures were also calculated for the agreement between the lumbar spine DXA AP and REMS T-scores, the lumbar spine DXA LL and REMS T-scores, and the femoral neck DXA vs REMS T-scores.

A repeated measures one-way Analysis of variance (ANOVA) with Greenhouse–Geisser correction in the case of violation of sphericity assumption (as assessed through Mauchly’s test), with post hoc analysis (Bonferroni) adjustment was used to compare the BMD data and fracture risk estimates acquired with DXA and REMS at the lumbar site (DXA AP and LL and REMS scans). A two-sided paired samples Student’s t-test was used to compare the T-scores, Z-scores and FRAX^®^ and DeFRA^®^ values at the total hip and femoral neck measured with DXA and REMS.

Differences in the AOCs score in the subgroup with and without vertebral fractures were tested through the Mann–Whitney *U* test. Spearman’s rho was run to explore correlations between AOCs score and the REMS and DXA T-scores.

Two-sided *p* values of 0.05 or less were considered to be statistically significant. Data were analysed using SPSS software, Version 22 (SPSS, Inc., Chicago, IL, USA).

## Results

We enrolled 41 patients. The anthropometric characteristics of the sample, biochemical parameters, and the data on fractures and medications are reported in Table 1.

**Table 1:** Anthropometrics, clinical and biochemical characteristics of the enrolled sample

Sample size (males:females)	41 (29:12)
Age (y)	
Mean (SD)	61.1 (13.7)
Median [IQR]	62 [52–73]
Min–Max	22–84
Height (cm)	
Mean (SD)	170 (9.5)
Median [IQR]	170 [165–176]
Min.–Max.	150–189
Body weight (Kg)	
Mean (SD)	73.7 (16.0)
Median [IQR]	74 [61–83]
Min.–Max.	50–107
Body mass index (Kg/m^2^)	
Mean (SD)	25.4 (4.5)
Median [IQR]	25 [22–27.8]
Min.–Max.	17.6–42.3
Aortic calcifications score	
Median [IRQ]	2 [0–6]
Min.–Max.	0–20
Disease duration—CKD (months)	
Mean (SD)	161 (139)
Median [IQR]	132 [48–140]
Min.–Max.	3–212
Time from CKD diagnosis to PD (months)	
Mean (SD)	142 (128)
Median [IQR]	125 [23–314]
Min.–Max.	2–617
Dialysis duration (months)	
Mean (SD)	19 (22)
Median [IQR]	10 [3–24]
Min.–Max.	1–86
S-calcium (mg/dL)	
Mean (SD)	8.9 (0.72)
Median [IQR]	9.1 [8.6–9.4]
Min.–Max.	6.6–10.4
S-phosphorous (mg/dL)	
Mean (SD)	5.38 (1.35)
Median [IQR]	5.4 [4.6–6.4]
Min.–Max.	2.66–7.89
PTH (pg/mL)	
Mean (SD)	41.0 (32.3)
Median [IQR]	31.4 [22.8–46.8]
Min.–Max.	10.3–172
Hyperparathyroidism	
PTH > 70 pg/mL	6%
25OH vitamin D (nmol/L)	
Mean (SD)	51 (18)
Median [IQR]	53 [36–72]
Min.–Max.	18.5–91.6
Hypovitaminosis D	
(< 50 nmol/L)	37.1%
BAP (μg/L)	
Mean (SD)	14.8 (8.2)
Median [IQR]	13 [9–17.5]
Min.–Max.	5–42
High BAP	
(> 22 μg/L)	17%
Patients with morphometric fractures (VFA)	15%
Patients with femoral fractures	2.4%
Total *n*° of morphometric fractures	12
Patients supplemented with D3	70%
Monthly D3 supplementation (median, IQR) (Iu)	25,000 [0, 0–30]
Patients treated with calcimimetics	10%
Patients treated with calcitriol	70%
Patients treated with chronic corticosteroid	2.4%
Patients treated with antiresorptives	0%

*CKD* chronic kidney disease, *PTH* parathyroid hormone, *ALP* alkaline phosphatase, *BAP* bone-specific alkaline phosphatase, *SD* standard deviation, *IQR* interquartile range, *PD* peritoneal dialysis, *VFA* vertebral fracture assessment

### T-scores and Z-scores comparisons

**Fig. 1:**
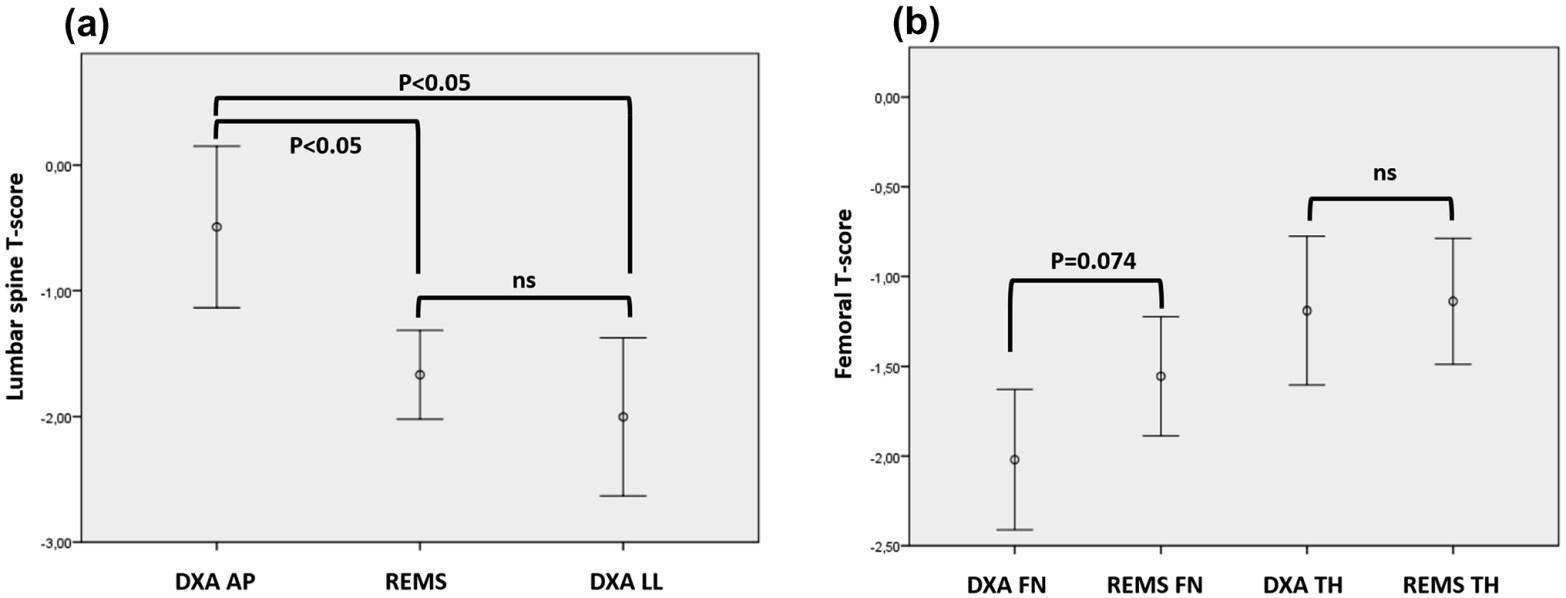
Comparison of the *T*-scores (error bars represent 95% CI) obtained with DXA and REMS at the lumbar spine (**A**; one-way ANOVA for repeated measures with Bonferroni adjustment), and at the femur (**B**, Student’s *t*-test for paired samples). *AP* anteroposterior, *LL* latero-lateral, *TH* total hip, *FN* femoral neck, 95% CI 95% confidence interval

The mean values with 95% confidence intervals (CI) of the *T*-scores at the AP and LL lumbar spine measured by DXA and REMS are depicted in Fig. 1A, while the *T*-scores at the femoral neck and total hip are reported in Fig. 1B. At the lumbar spine, we found a statistically significant difference between the *T*-score of the AP DXA scan and both the LL DXA and REMS, while no difference was found between the LL DXA and REMS scan. No statistically significant difference was found between the DXA and REMS *T*-scores at either the femoral neck or total hip.

When all sites were considered, 51.3% of the patients satisfied the criterion for densitometric osteoporosis when measured by DXA (the prevalence decreased to 43.6% when the LL scan was excluded) and 32.4% with REMS.

At the lumbar spine, the mean DXA AP *Z*-score was 0.08 ± 1.16, the mean DXA LL Z-score was − 0.62 ± 1.70, while the mean REMS *Z*-score was − 0.75 ± 0.78.

The one-way repeated measures ANOVA among the tree *Z*-scores at the lumbar spine (DXA AP, DXA LL and REMS) resulted statistically significant (*p* = 0.006), and at the post-hoc analysis we found significant differences was between the AP *Z*-score and the REMS-*Z*-score (*p* = 0.004) and between the DXA AP *Z*-score and LL *Z*-score (*p* = 0.002).

At the femoral neck, the mean DXA *Z*-score was − 0.78 ± 1.01, while the mean REMS *Z*-score was − 0.63 ± 0.65, *p* = NS. At the total hip, the mean DXA *Z*-score was − 0.51 ± 1.0, while the mean REMS *Z*-score was − 0.74 ± 0.68, *p* = NS. When all sites were considered, 15.4% of the patients satisfied the criterion for BMD below the expected when measured by DXA (12.8% when the LL measurement were excluded), and 7% with REMS.

### TBS

The mean TBS was 1.290 ± 0.146 (min–max: 0.985–1.583). The TBS *T*-score was correlated with the T-scored measured through DXA at all sites (AP lumbar spine *R*^2^ = 0.31, *p* < 0.01; LL lumbar spine *R*^2^ = 0.21, *p* < 0.01; femoral neck *R*^2^ = 0.27, *p* < 0.01; total hip *R*^2^ = 0.31, *p* < 0.01), while was not correlated with the REMS T-score at any site. TBS was not significantly different between fractured and non-fractured subjects and was moderately negatively correlated with the AOCs score (Spearman’s rho − 0.407, *p* = 0.01). TBS T-score was not correlated with disease duration.

### Risk assessment tools comparison

**Fig. 2:**
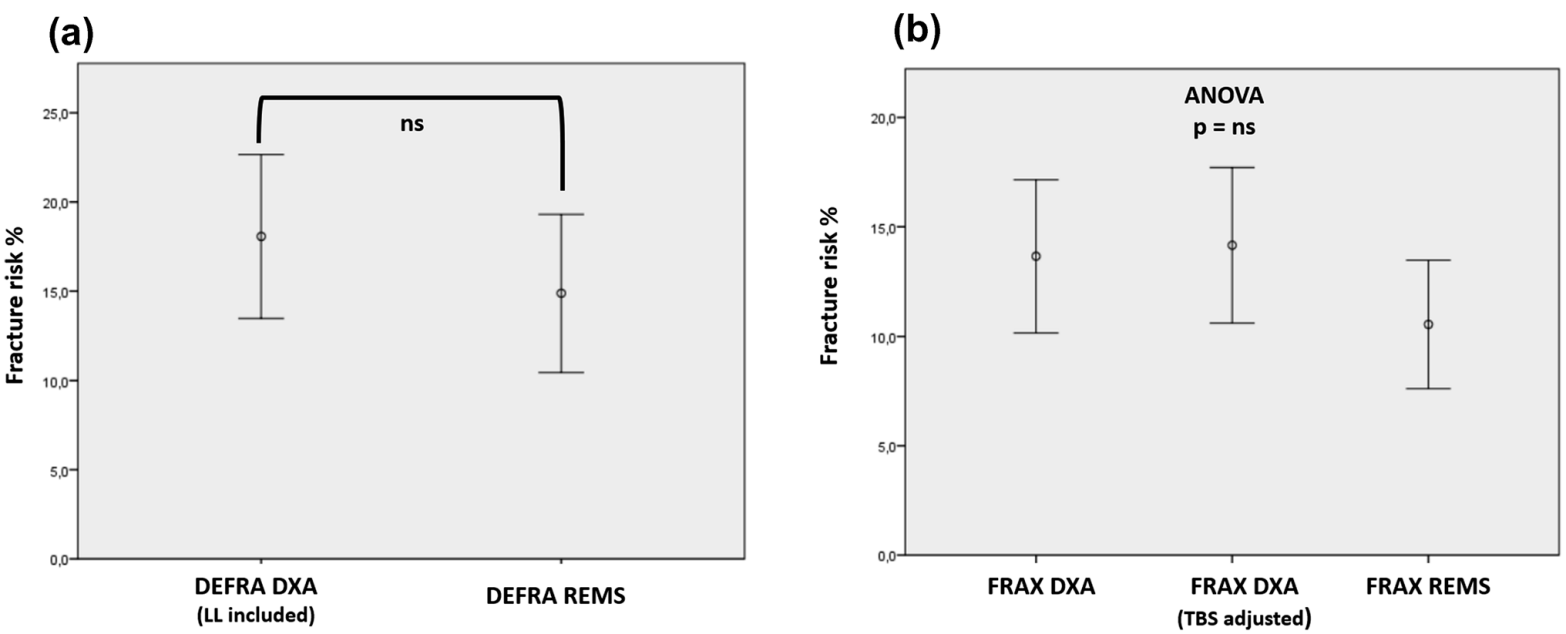
Comparison between the DeFRA^®^ DXA and REMS-derived outputs **A** and FRAX^®^ DXA and REMS-derived outputs **B** raw and after correction for TBS. *DeFRA, FRAX*-derived risk assessment tool, *FRAX* Fracture Risk Assessment tool, *AP* anteroposterior, *LL* latero-lateral, *TH* total hip, *FN* femoral neck, *TBS* trabecular bone score

No statistically significant differences in the DeFRA^®^ or FRAX^®^ outputs (both raw and TBS-adjusted) were found when calculated upon the data from DXA or REMS (Fig. 2A, B, respectively).

### Agreement measures

The Cohen’s *k* correlation coefficients for the diagnosis of densitometric osteoporosis were, at the lumbar spine, between REMS and LL DXA: 0.321, *p* = 0.026 (fair agreement). For REMS LS and AP DXA: 0.19, *p* = NS. At the femoral neck: 0.445, *p* < 0.01 (moderate agreement) and at the total hip 0.784, *p* < 0.001 (substantial agreement). When we tested the agreement after considering the worst T-score among all the different sites: 0.421, *p* < 0.001 (moderate agreement).

The Cohen’s *k* correlation coefficient for the diagnosis of BMD below the expected (worst site considered) between DXA and REMS was 0.633, *p* < 0.01 (substantial agreement).

The calculated ICC were in line with the Cohen’s *k* analysis, showing the strongest association between DXA and REMS at the femoral neck and the weakest association at the lumbar spine between the AP DXA and the REMS measurements (Supplementary appendix).

No significant difference was found in the AOCs score between the fractured and non-fractured subjects.

### Correlations between BMD (DXA and REMS-measured) and AOCs

**Fig. 3:**
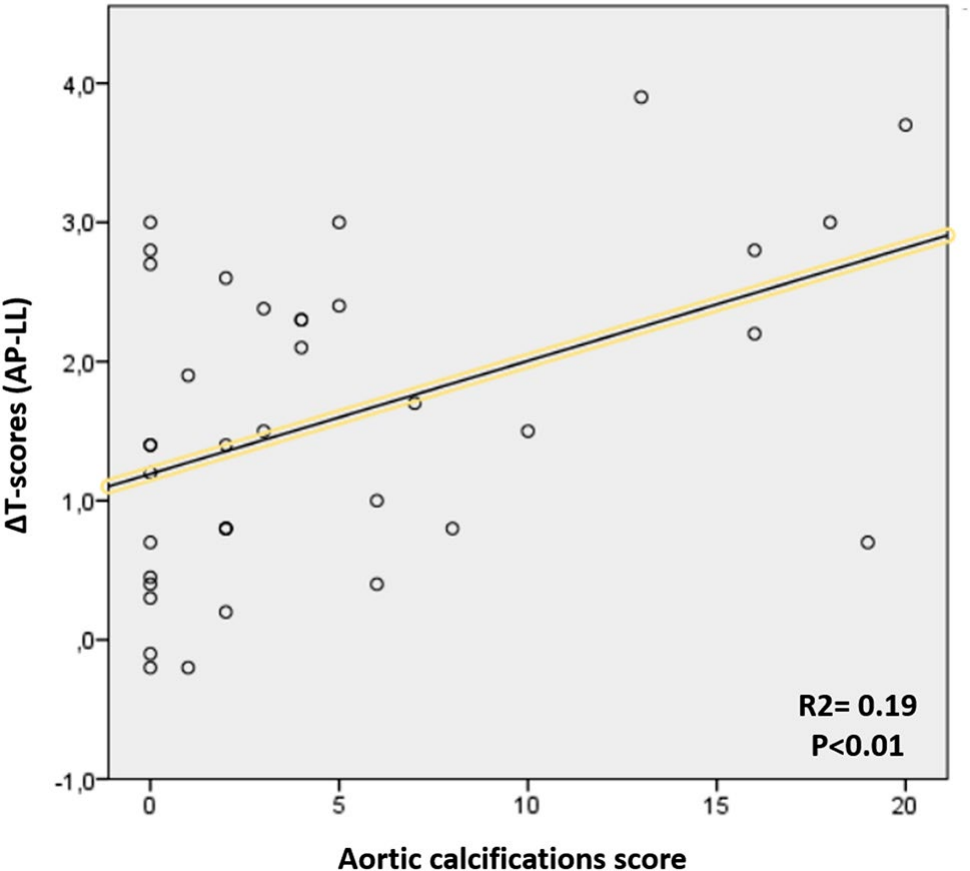
Scatter plot reporting the relationship between the difference between the AP and LL *T*-scores (∆ *T*-scores AP-LL; *y*-axis) and the aortic calcifications score (*x*-axis). Significance and R2 refer to the Pearson’s correlation

We found a statistically significant positive correlation of moderate strength between the total calcification score and the difference between the DXA AP *T*-score and the DXA LL *T*-score at the lumbar spine (*p* < 0.01, Spearman’s rho correlation coefficient = 0.402), Fig. 3. On the other hand, we found a significant negative correlation between the total AOCs score and the *T*-score at all sites (both DXA and REMS); the only exception was for the AP DXA (Table 2).

**Table 2:** Correlations between the total calcification score and the *T*-scores measure by DXA and REMS

Lumbar spine			
	DXA Ts AP	DXA Ts LL	REMS Ts
Calcification score			
Spearman’s correlation coefficient	− 0.175	− 0.359	− 0.438
Sig.	NS	*P* = 0.027	*P* = 0.011

Proximal femur				
	DXA Ts TH	DXA Ts FN	REMS Ts TH	REMS Ts FN
Calcification score				
Spearman’s correlation coefficient	− 0.527	− 0.523	− 0.383	− 0.485
Sig.	*P* < 0.01	*P* < 0.01	*P* < 0.01	*P* = 0.028

*AP* anteroposterior, *LL* latero-lateral, *TH* total hip, *FN* femoral neck

## Discussion

The present study showed for the first time a promising agreement between DXA and REMS in the diagnosis of densitometric osteoporosis in a real-life sample of PD patients. Moreover, these data also reported comparable results between the fracture risk estimates derived by the two technologies. Furthermore, our data supports the robustness of the REMS technique against the influence of AOCs when assessing the lumbar spine BMD, as already suggested in recent case series [19] and in a cross-sectional study [20]. On the contrary, as already shown in previous studies [10, 11], AOCs predispose to an artefactual increase in lumbar spine BMD when measured with the commonly adopted anteroposterior approach, with the consequent risk of obtaining a misleading T-score value. A possible solution for this problem could be to turn the patient on the side and switch to a LL scan (thereby bypassing the aorta), as already suggested in other special populations characterised by axial ectopic ossification [12]. However, this is still uncommon in the daily clinical practice; not all densitometers have this feature, and the history of CKD may be overlooked by the technician performing the exam. For these reasons, REMS may help overcome these limitations in special populations such as CKD patients.

In addition, the positive correlation between the calcification score and the DXA LL-AP T-scores discrepancy supports the role of AOCs as culprits for the artefactual BMD overestimation with DXA.

Though we did not observe a significant difference in the calcifications score between the fractured and non-fractured subjects, arguably because of underpowered sample size, we did find a significant negative correlation between the calcification score and the BMD measured with both the imaging techniques. Our data therefore confirms the association between systemic skeletal involvement and AOCs also in patients undergoing PD. This finding is in line with other clinical data reporting the worsening of CKD-MBD with the severity of the kidney damage and its disease duration [21].

Future studies and longitudinal data should focus on specifically testing this hypothesis and assessing the sensitivity to change (over time and after treatment with bone acting agents) of the REMS technique and comparing it to the current gold standard.

Interestingly, we confirmed a negative correlation between the AOCs score and TBS. This is in line with a previous study on dialysis patients that used similar methods [22], and corroborates the potential usefulness of techniques assessing bone quality in CKD patients.

Finally, in our cohort, only a minority of patients (6%, *N* = 2) patients showed increased levels of PTH. This is somewhat difficult to explain, as more than 50% subjects receiving PD usually show increased PTH serum concentrations [23]. Presently, we do not think that this observation could have significantly influenced our findings in terms of imaging comparisons, though we warrant further studies to investigate the relationship between serum and imaging biomarkers of osteometabolic health in CKD/PD patients.

Our study has its limitations. First, we emphasize that this is an exploratory study with a limited sample size, not sufficient to run a validation process, and the absence of a control group represents a major limitation. In addition, the AOCs score adopted was originally studied for X-rays and not DXA. However, previous studies already applied it to LL DXA evaluation, with good correlation coefficients with X-rays [24] and reproducibility data in patients receiving haemodialysis [25]. Clearly, longitudinal designs are needed to scrutinize the REMS sensitivity to change over time and after treatment.

In conclusion, this study shows a promising agreement, in a real-life PD setting, between the DXA and REMS BMD values and in the consequent fracture risk assessment. The availability of a novel technique for the assessment of BMD, characterised by nimble machinery, absence of ionising radiations and good robustness to measurement artifacts could be extremely useful in the everyday clinical practice.

## Supplementary Information

Below is the link to the electronic supplementary material.

Supplementary file 1 (DOCX 14 KB)

Supplementary file 2 (TIF 343 KB)

## Data Availability

The data that support the findings of this study are available from the corresponding author upon reasonable request.
