# Supplementary material for: Radiofrequency echographic multi-spectrometry and DXA for the evaluation of bone mineral density in a peritoneal dialysis setting
Source: Aging Clin Exp Res. 2022 Nov 3;35(1):185–92. doi: 10.1007/s40520-022-02286-7 (PMC9816283; doi:10.1007/s40520-022-02286-7)
Supplement: Supplementary file 1 — (DOCX 14 KB) [file 40520_2022_2286_MOESM1_ESM.docx]

**Supplementary appendix**

**Intraclass correlation coefficient (ICC)**

**Table 1**: ICC Calculation results of T-score lumbar spine lumbar spine (LS) antero-posterior (AP) values obtained with both DXA and REMS, using single measures and average measures, absolute agreement, the same raters for all subjects, 2-way model.

|  | Intraclass correlation^a^ | 95% Confidence Interval |
| --- | --- | --- |
| Single measures^b^ | 0.35 | 0.004134 to 0.6218 |
| Average measures^c^ | 0.52 | 0.008234 to 0.7668 |

**Table 2**: ICC Calculation results of T-score LS latero-lateral (LL) values obtained with DXA and T-score LS obtained with REMS, using single measures and average measures, absolute agreement, the same raters for all subjects, 2-way model.

|  | Intraclass correlation^a^ | 95% Confidence Interval |
| --- | --- | --- |
| Single measures^b^ | 0.42 | 0.1019 to 0.6659 |
| Average measures^c^ | 0.60 | 0.1849 to 0.7994 |

**Table 2**: ICC Calculation results of T-score femoral neck values obtained with both DXA and REMS, using single measures and average measures, absolute agreement, the same raters for all subjects, 2-way model.

|  | Intraclass correlation^a^ | 95% Confidence Interval |
| --- | --- | --- |
| Single measures^b^ | 0.55 | 0.2466 to 0.7517 |
| Average measures^c^ | 0.71 | 0.3956 to 0.8582 |

^a^ The degree of absolute agreement among measurements.

^b^ Estimates the reliability of single ratings.

^c^ Estimates the reliability of averages of *k* ratings.

The value of an ICC can range from 0 to 1, with 0 indicating no reliability among raters and 1 indicating perfect reliability. Based on the 95%confident interval of the ICC estimate, values less than 0.5, between 0.5 and 0.75, between 0.75 and 0.9, and greater than 0.90 are indicative of poor, moderate, good, and excellent reliability, respectively.
