# Supplementary figures and images for: Radiofrequency echographic multi-spectrometry and DXA for the evaluation of bone mineral density in a peritoneal dialysis setting
Source: Aging Clin Exp Res. 2022 Nov 3;35(1):185–92. doi: 10.1007/s40520-022-02286-7 (PMC9816283; doi:10.1007/s40520-022-02286-7)

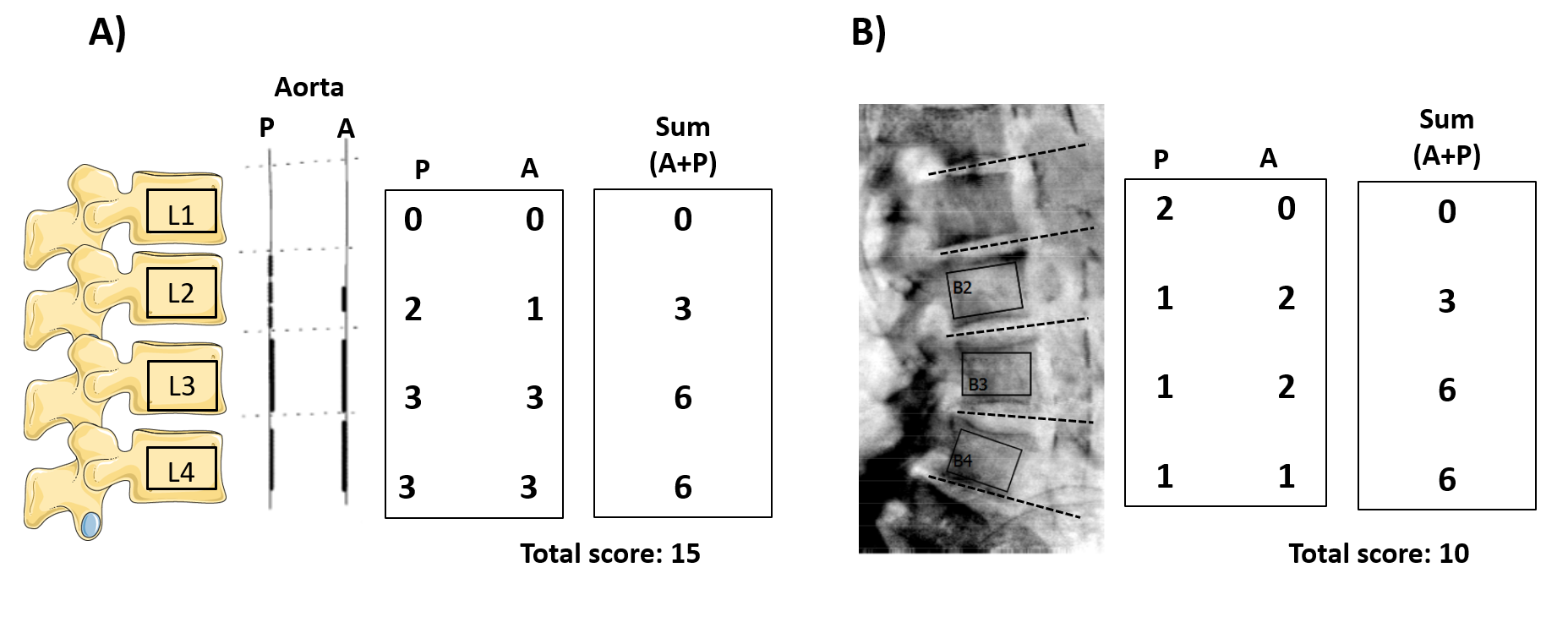

Supplement: Supplementary file 2 — (TIF 343 KB) [file 40520_2022_2286_MOESM2_ESM.tif]
